# Metabotropic glutamate receptors modulate exocytotic tau release and propagation

**DOI:** 10.1124/jpet.122.001307

**Published:** 2022-09-18

**Authors:** Francesca Mazzo, Ioana Butnaru, Olivera Grubisha, Elena Ficulle, Helen Sanger, Griffin Fitzgerald, Feng Pan, Francesca Pasqui, Tracey Murray, James Monn, Xia Li, Michael Hutton, Suchira Bose, Giampietro Schiavo, Emanuele Sher

**Affiliations:** Eli Lilly & Co Ltd, Neuroscience, 8 Arlington Square West, Downshire Way, Bracknell RG12 1PU, UK; UK Dementia Research Institute at UCL, University College London, London WC1E 6BT, UK; Eli Lilly & Co Ltd, Neuroscience, 8 Arlington Square West, Downshire Way, Bracknell RG12 1PU, UK; Eli Lilly & Co Ltd, Neuroscience, 8 Arlington Square West, Downshire Way, Bracknell RG12 1PU, UK; Eli Lilly & Co Ltd, Neuroscience, 8 Arlington Square West, Downshire Way, Bracknell RG12 1PU, UK; Lilly Research Laboratories, Eli Lilly and Company, Indianapolis, IN 46285, USA; Lilly Research Laboratories, Eli Lilly and Company, Indianapolis, IN 46285, USA; Eli Lilly & Co Ltd, Neuroscience, 8 Arlington Square West, Downshire Way, Bracknell RG12 1PU, UK; Eli Lilly & Co Ltd, Neuroscience, 8 Arlington Square West, Downshire Way, Bracknell RG12 1PU, UK; Lilly Research Laboratories, Eli Lilly and Company, Indianapolis, IN 46285, USA; Lilly Research Laboratories, Eli Lilly and Company, Indianapolis, IN 46285, USA; Lilly Research Laboratories, Eli Lilly and Company, Indianapolis, IN 46285, USA; Eli Lilly & Co Ltd, Neuroscience, 8 Arlington Square West, Downshire Way, Bracknell RG12 1PU, UK; UK Dementia Research Institute at UCL, University College London, London WC1E 6BT, UK; Dept. of Neuromuscular Diseases, Queen Square Institute of Neurology, University College London, Queen Square House, London WC1N 3BG, UK; Eli Lilly & Co Ltd, Neuroscience, 8 Arlington Square West, Downshire Way, Bracknell RG12 1PU, UK

**Keywords:** Alzheimer’s disease, botulinum neurotoxin, brain, SNAP25, synapse

## Abstract

Using synaptosomes purified from the brains of two transgenic mouse models overexpressing mutated human tau (TgP301S and Tg4510) and brains of patients with sporadic Alzheimer’s disease, we showed that aggregated and hyperphosphorylated tau was both present in purified synaptosomes and released in a calcium- and SNAP25-dependent manner. In all mouse and human synaptosomal preparations, tau release was inhibited by the selective mGlu2/3 receptor agonist LY379268, an effect prevented by the selective mGlu2/3 antagonist LY341495. LY379268 was also able to block pathological tau propagation between primary neurons in an *in vitro* microfluidic cellular model. These novel results are transformational for our understanding of the molecular mechanisms mediating tau release and propagation at synaptic terminals in Alzheimer’s disease and suggest these processes could be inhibited therapeutically by the selective activation of presynaptic G-protein-coupled receptors.

## Introduction

Tauopathies share as a common feature the intraneuronal accumulation of pathological (hyperphosphorylated and aggregated) tau, which defines a wide range of neurodegenerative diseases associated with dementia (Spillantini and Goedert, 2013; Gotz et al., 2019). Pathological tau accumulation in these tauopathies has been proposed to follow specific spatiotemporal patterns of neurocircuit spreading, with the sequence of brain regions impacted driving symptoms and clinical progression of each disease (Colin et al., 2020). In Alzheimer’s disease, this spreading hypothesis was supported at first by post-mortem pathological examinations (Braak and Braak, 1991; Spillantini and Goedert, 2013; Braak and Del Tredici, 2016; Wang and Mandelkow, 2016; Goedert et al., 2017). More recently, evidence of this process in living patients has come through positron emission tomography (PET) imaging with novel tau tracers, which has further confirmed that tau propagation correlates with cognitive worsening (Johnson et al., 2016; Scholl et al., 2016; Franzmeier et al., 2019; Vogels et al., 2020).

Different mechanisms for the spreading of tau pathology in the nervous system have been proposed, but a dominant view is that pathological tau undergoes cell-to-cell transfer, at least in part via synaptic contacts (Clavaguera et al., 2009; de Calignon et al., 2012; Liu et al., 2012; Ahmed et al., 2014; Lewis and Dickson, 2016; Wang and Mandelkow, 2016). Whilst recent findings highlight the role of tau in synaptic physiology and dysfunction (Zhou et al., 2017; McInnes et al., 2018), understanding how pathological tau reaches nerve terminals, is released, and eventually taken up by postsynaptic cells (De La-Rocque et al., 2021) will be critical for developing pharmacological interventions that target these currently untreatable diseases.

Accumulating lines of evidence indicate that both normal and pathological tau are released by neurons in an activity-dependent manner. To date, most of these studies have been conducted in rodent models, both *in vitro* (Pooler et al., 2013; Croft et al., 2017) and *in vivo* (Yamada et al., 2014). Furthermore, the presence of different forms of tau (Fein et al., 2008; Tai et al., 2014) and its release (Sokolow et al., 2015) have also been documented in human synaptosomes. However, the molecular mechanisms controlling tau release are not yet completely elucidated. In particular, the subcellular localization and specific vesicular compartments (e.g., synaptic vesicles, large dense-core granules, exosomes, and other membrane-bound organelles) carrying pathological tau at synaptic terminals, the specific components forming the tau release machinery (e.g., SNARE proteins and their regulators), and the calcium-dependency of the process, are all areas of intense investigation. The paucity of molecular information regarding pathological tau dynamics ultimately determines the lack of effective strategies to inhibit pharmacologically these processes efficiently and safely.

Here, using synaptosomes purified from the brains of two independent strains of transgenic mice overexpressing mutant human tau - TgP301S (Allen et al., 2002) and Tg4510 (Santacruz et al., 2005) -, and from the brains of patients with sporadic Alzheimer’s disease, we demonstrate that hyper-phosphorylated and aggregated tau is not only present in isolated synaptic terminals, but that it is also released in a depolarisation- and calcium-dependent manner. Pathological tau release relies on the integrity of the synaptic SNARE protein SNAP25, since specific cleavage of this essential component of the exocytotic machinery by botulinum neurotoxin A (BoNT/A) significantly inhibits hyperphosphorylated and aggregated tau secretion. Furthermore, we found in all three preparations that similarly to other neurotransmitter release processes, pathological tau release is inhibited by activation of presynaptic mGlu2/3 receptors.

## Materials and methods

### Animals

All animal procedures were performed in accordance with the Animals (Scientific Procedures) Act 1986 and were approved by the Eli Lilly Animal Welfare Board. Tg4510 mice, male, were used at 24 weeks; TgP301S, male, at 22 weeks; C57 black/6J mice, male, at 26 weeks. For slice electrophysiology, 5 weeks-old-male Sprague Dawley rats were used.

### Brain samples

Frozen fragments of human normal and Alzheimer’s disease frontal cortex and hippocampus were obtained with ethical approval from the Oregon Alzheimer’s Disease Center and UCL Brain Bank, kept frozen at -80°C and utilized according to the Code of Ethics of the World Medical Association (Declaration of Helsinki, 1964).

### Drugs

Ionomycin from Tocris (Bristol, UK) was freshly prepared in DMSO and then diluted in Krebs buffer (containing 140 mM NaCl, 3 mM KCl, 1.2 mM MgCl_2_, 1.2 mM NaH_2_PO_4_, 5 mM NaHCO_3_, 10 mM glucose, and 10 mM HEPES-NaOH, pH 7.4) at a final concentration of 100 nM. LY379268 (Monn et al., 1999; Sanger et al., 2013) and LY341495 (Johnson et al., 1999) were synthesized at the Lilly Research Laboratories (Indianapolis, USA). LY379268 was dissolved in distilled water, whereas LY341495 stocks were prepared in NaOH 0.1 M.

### Antibodies

Anti-cleaved SNAP25 antibody (Antonucci et al., 2008) was a kind gift from Ornella Rossetto (University of Padua, Italy). The following antibodies were kind gifts from Peter Davies (Albert Einstein College of Medicine, New York): total tau: DA9 (aa 102–140) (Tremblay et al., 2010), PG5 (pSer409) (Jicha et al., 1999), PHF1 (pS396/404) (Greenberg et al., 1992) and CP27 (human tau aa 130-150) (Petry et al., 2014), whereas the conformation-dependent tau antibody MC1 (epitope within aa 312-322) (Jicha et al., 1997) was purchased from Thermo Fisher Scientific. Phosphorylation-dependent anti-tau antibodies AT8, (pS202/pT205), AT100 (pS212/pT214), phosphorylation-independent antibody HT7 (aa 159–163) and anti-tubulin antibody were also from Thermo Fisher Scientific. The rodent-specific tau antibody T49, was purchased from Merck (MABN827). The anti-GADPH antibody was from Life Technologies; anti-GFAP, anti-myelin basic protein, anti-synaptophysin and anti-LDH antibodies from Abcam; Iba1 from Wako; anti-PSD95 and anti-GluN2B from BD Biosciences; anti-total SNAP25 from BioLegend; anti-VAMP2 from SYSY Systems. Primary antibodies used for STED were as follows: synaptophysin (mouse; MAB5258, Chemicon), SNAP25 (rabbit; ab5666, Abcam), PSD-95 (rabbit; 18258, Abcam), pathological tau (biotinylated MC1; Eli Lilly), phosphorylated tau (biotinylated AT8; Eli Lilly). Secondary probes for STED included: donkey anti-mouse AlexaFluor594-conjugated antibody (150108, Abcam), goat anti-mouse AlexaFluor488-conjugated antibody (a11008, Invitrogen), and streptavidin STAR635 (human samples; 1-1301-002-6, Abberior), and streptavidin Dylight 549 (mouse samples; SA-5549, Vector).

### Purification of synaptosomes and total lysate from rodent and human brain

Mouse TgP301S and Tg4510 and human normal and Alzheimer’s disease brains were homogenized in 10 volumes of 0.32 M sucrose, 10 mM EDTA, 50 mM Tris-HCl pH 7.4, containing protease (Roche) and phosphatase (Millipore) inhibitors. The homogenate was first centrifuged at 1,500 *g* for 15 min and the lysate collected. In different sets of experiments, synaptosomes were purified with either a sucrose gradient or with a Percoll gradient, as previously described (Mazzo et al., 2016).

### Sarkosyl and Triton X-100 solubility

Detergent insoluble and soluble fractions were separated as previously described (Katsikoudi et al., 2020). An aliquot of synaptosomes or supernatant after release was incubated with sarkosyl (1% final volume) or Triton X-100 (1.5%) for 1 h at room temperature under shaking, before centrifugation at 150,000 *g* for 1 h at 4°C. The pellet, containing detergent-insoluble tau and the supernatant, containing detergent-soluble tau were then resolved by western blotting.

### Western blotting in reducing and non-reducing conditions

Samples were prepared in Laemmli buffer with (reducing) or without (non-reducing) β-mercaptoethanol (BME), heated at 95°C for 3 min and run in NuPageR™ Novex 4-12% Bis-Tris midi gels under reducing conditions, followed by transfer onto nitrocellulose membranes (Amersham Protran Premium; 0.45 µm) using a Mini Trans-Blot Electrophoretic Transfer Cell (Bio-Rad) and probing with AT8 or HT7 antibodies (Mazzo et al., 2016). For reducing conditions, samples were loaded on 4-15% Mini-PROTEAN TGX gels (Bio-Rad) in Tris-glycine-SDS buffer (Bio-Rad). Membranes were incubated with either 5% (w/v) non-fat dry milk (Sigma) or bovine serum albumin (BSA; Sigma) dissolved in Tris-Buffered Saline with 0.1% Tween 20 (TBS-T; Sigma) for 45 min at room temperature. Membranes were briefly rinsed with TBS and incubated with the primary antibodies diluted in TBS-T overnight at 4°C. After washing in TBS-T, membranes were further incubated for 1 h at room temperature with the corresponding horseradish peroxidase (HRP) -conjugated secondary antibodies (Abcam, DAKO) diluted in blocking buffer (5% non-fat dry milk in TBS-T). Membranes were incubated for 1 min at room temperature with Immobilon Crescendo HRP Substrate (Millipore), imaged with an Amersham 680 Imager and optical density measurements analysed with ImageJ.

### Sandwich ELISA

Plates were coated with 2 mg/ml DA9 as a capture antibody. Following blocking with BB3 SynBlock buffer (Immunochemistry Technologies) at room temperature for 2 h, plates were incubated with samples overnight at 4°C. Following washing, tau was detected using biotinylated CP27 + HT7 (for total tau), or AT8 (for phospho-tau), and subsequently with HRP-linked streptavidin (Invitrogen). Plates were incubated with TMB substrate, the reaction stopped with 2 M sulphuric acid, and read at 450 nm on a plate reader (Molecular Devices).

### Cortical neurons seeding and tau aggregation assay

Rat embryonic cortical neurons (RCN) were plated at 125,000 cells/cm^2^ in Neurobasal (Invitrogen) supplemented with 0.4 mM L-glutamine (Life Technologies), MACS Neurobrew-21 (Miltenyl Biotech), and 50 U/ml pen-strep. Cells were transduced with adeno-associated virus 2 (AAV2) expressing eGFP-P2A-MAPT P301S under the human synapsin 1 promoter (Vector Biolabs). MAPT was the 1N4R isoform (NM_001123067.3) encoding the P301S mutation. Cells were plated, transduced on day *in vitro* (DIV) 0 and then treated on DIV8 with purified TgP301S synaptosomes (17 µg/ml protein) or the sarkosyl-insoluble synaptosome release fraction. Samples were sonicated 5 times for 1 s prior to addition to cells. After 24 h incubation, the media was removed. On DIV14, RCN were fixed with 4% paraformaldehyde for 15 min and processed using a standard immunocytochemistry protocol. Primary antibodies, MC1 or PG5 and CP27 were detected with goat anti-mouse IgG1 AlexaFluor568 or IgG3 AlexaFluor594 and IgG2b AlexaFluor647, respectively. Images were taken on the Operetta CSL system (Perkin Elmer) and analysed using the Harmony 6.0 software. Briefly, nuclei within the GFP region were detected and expanded to encompass the cell body. Maximum MC1 and CP27 intensity per cell body was measured and a threshold MC1 and PG5 intensity was set based on untreated control. Data represent the mean +/- SD of 3 independent experiments, each performed in triplicate.

### Preparation of synaptosomes for synaptic release

Crude synaptosomes for tau release experiments were isolated as previously described (McMahon et al., 1992). Briefly, mouse forebrains or Alzheimer’s disease frontal cortex (600-800 mg, Braak stage VI) were fast thawed at 37°C for 80 s and transferred on ice. All subsequent steps were carried out at 4°C, if not otherwise specified. Tissues were homogenized with a Dounce tissue grinder in homogenization buffer (0.32 M sucrose, 10 mM EDTA, 50 mM Tris-HCl, pH 7.4 with protease and phosphatase inhibitors), and the homogenates centrifuged at 1,500 *g* for 15 min. To collect the P2 pellet fraction, the supernatant S1 was centrifuged at 16,000 *g* for 20 min. P2 pellet was resuspended at 5 mg/ml in homogenization buffer, split into equal samples and further centrifuged at 16,000 *g* for 10 min. Pellets were resuspended in Krebs buffer and centrifuged at 16,000 *g* for 5 min. Synaptosomes were gently resuspended and then pre-incubated in Krebs buffer containing either 100 nM of BoNT/A or 100 nM BoNT/D for 30 min at 37°C (Schiavo et al., 1993), or 1 µM mGluR2/3 agonist LY379268 and 10 µM antagonist LY341495 for 15 min at 4°C. Samples were then centrifuged for 3 min at 16,000 *g*, the supernatant was removed and pellets were resuspended in Krebs buffer containing either 100 nM ionomycin or 50 mM KCl and incubated for 30 min at 37°C to allow tau release. Supernatants were collected following centrifugation at 16,000 *g* for 3 min. For human samples, the supernatants were further incubated with 1% sarkosyl, shaken 1 h at room temperature and centrifuged at 150,000 *g* for 1 h. Sarkosyl insoluble pellets were resuspended in Dulbecco’s Phosphate Buffered Saline (D-PBS). 4x Laemmli sample buffer (BioRad) containing BME was added to the samples, which were then boiled for 5 min at 95°C.

### Slice electrophysiology

5 weeks-old-male Sprague Dawley rats were anaesthetized by isoflurane and killed by decapitation. Transverse hippocampal slices (400 μm) were prepared as described (Witkin et al., 2017), then transferred in a submerged holding chamber containing artificial cerebrospinal fluid (aCSF) at room temperature and allowed to recover for at least 1 h. Recordings were performed under continuous aCSF perfusion (3.5 ml/min) at 31.5°C. Recording and stimulation electrodes were positioned in the dentate gyrus and the medial perforant path (MPP), respectively, to measure synaptic transmission from the entorhinal cortex to the dentate gyrus (EC>DG pathway). The maximal amplitude of the field excitatory postsynaptic potential (fEPSP) was used as a measure of synaptic strength and stimulus intensity was set to a level eliciting the biggest response achievable without having the amplitude contaminated by a population spike. fEPSPs were evoked every 30 s and averages of 4 successive trials were captured. Correct positioning of the electrodes was verified by application of a paired-pulse (PP) protocol: application of PP at an inter-stimulus interval of 50 ms induces PP depression in the MPP. Thus, the effect of PP stimulation was assessed, and electrode position was accepted only when PP depression was displayed. Stable responses were obtained for at least 40 min before the agonist LY379268 (1 μM) was applied for at least 20 min. In parallel experiments, stable responses were obtained for at least 20 min before the antagonist LY341495 (10 μM) was applied for 20 min alone, after which it was co-applied with the agonist LY379268 for further 20 min. Responses were normalised to their respective baseline; all data were expressed as mean ± SEM. The difference between treatment groups was assessed using an unpaired Student’s t test comparing the average of the last 5 points (10 min) during the final applications.

### Stimulated emission depletion (STED) imaging

Samples for STED imaging were prepared as follows. Postmortem tissue samples from mice and AD patients were rinsed in ice-cold homogenizing buffer (HB; 320 mM sucrose, 1 mM EDTA, 5 mM Tris-HCl, pH 7.4), then homogenized using a glass/teflon grinder. The resulting lysate was subjected to Percoll gradient (23%, 15%, 10%, 3% Percoll in HB buffer) centrifugation, with resulting fractions F3-F4 collected and diluted in HB buffer. Fractionated pellets were resuspended in Krebs buffer. Synaptosomes were further diluted in PBS-MC (1 mM MgCl_2_, 1 mM CaCl_2_, pH 7.4), applied to poly-D-lysine coated chamber slides, and fixed in 1% paraformaldehyde for 10 min. Fixed synaptosomes were washed in PBS-MC, 3 × 5 min and permeabilized for 10 min in 0.1% Triton X-100 in PBS-MC.

Immunolabeling of fixed synaptosomes. Synaptosomes were blocked in 3% BSA in PBS-MC for 30 min, then incubated overnight in primary antibodies against synaptophysin, SNAP25, PSD-95, pathological tau (biotinylated MC1), and phosphorylated tau (biotinylated AT8). After washing, synaptosomes were then incubated in secondary probes (described above) for 1 h. Samples were washed then mounted in Prolong Diamond (Fisher, P36961). Dilutions were 1:50 for primary and 1:100 for secondary probes in 3% BSA/PBS-MC.

Fields containing synaptosomes, which were identified based on previously described criteria (Tai et al., 2014), were visualised via brightfield illumination. STED imaging was conducted in sequential scan format (TCS SP8 STED 3X, Leica Microsystems) using the following parameters: 594 nm excitation, 775 nm depletion (AlexaFluor594); 635 nm excitation, 775 nm depletion (STAR 635); 488 nm excitation, 592 nm depletion (AlexaFluor488). Acquired z-stacks were cropped to include 8 planes with optimal focus. Cropped z-stacks were corrected for thermal drift, then deconvolution was applied using the Classic Maximum Likelihood Estimation method (Huygens Professional, Scientific Volume Imaging). Deconvolution algorithm parameters were set as follows: SNR = 20, max iterations = 40, quality change threshold = 0.05.

Quantification and colocalization analysis. Colocalization analysis was conducted using Imaris (Bitplane). Brightfield images were used to disqualify voxels lying outside of synaptosomes boundaries. Voxel intensity within synaptosome boundaries was normalized for each fluorophore to occupy the full histogram range (0-255), then 3D median filtered (3 × 3 × 3 kernel). Colocalized voxels for fluorescent marker pairs were mapped by scatterplot thresholding (threshold > 1 for all fluorophore pairs). Thresholded voxels were counted, and percent colocalization was then calculated. This process was repeated for the entire dataset, with resulting output tabulated for statistical analyses (Prism 7, Graphpad). Significant differences in percent colocalization between marker pairings were analysed using independent samples *t*-test.

### Microfluidic cultures

Microfluidic devices (M. Zagnoni, Strathclyde University, UK) were plasma bonded on large cover glasses (50 × 75 mm; thickness No. 1) using an oxygen plasma cleaner (Henniker Plasma HPT-200; 60 s at 30% power, 4 sccm oxygen). The devices were poly-ornithine coated (Sigma, P4957), for 90 min at 37°C and plated with 5 µl of 28,000 cells/µl in RCN medium. Media was changed at DIV3 to remove non-adherent cells. Human Alzheimer’s disease seed (Greenberg and Davies, 1990) was diluted in RCN media, sonicated on ice for 1 min at 20% amplitude (60 × 1 s pulses) and filtered with Pal Acrodisc 20 µm. To create a hydrostatic gradient, cells were treated in the seeding side with 1 µg/ml of human Alzheimer’s disease seed by adding 30 µl of seed solution, and 60 µl of either the mGlu2/3 agonist LY379268, the mGlu2/3 antagonist LY341495, or both (final concentration of 10 µM in media) on the propagation side. At DIV10 and 17, the medium was completely removed and replenished with media only on the seeding side (30 µl) and with the treatment at the propagation side (60 µl). At DIV21, cells were fixed with ice-cold methanol to remove soluble proteins, and immunofluorescence was performed overnight using T49 primary antibody at 1:1,000 diluted in blocking buffer (Intercept blocking buffer with 0.1% Triton X-100). Goat anti-mouse AlexaFluor647-conjugated IgG1 secondary antibody (Life Technologies) with 1:1,000 Hoechst in blocking buffer was added for 1 h at room temperature. Plates were imaged with the Opera Phenix and analyzed with the Harmony Software and the Cell Counter as previously described (Katsikoudi et al., 2020).

## Results

### Calcium-dependent release of aggregated tau

**Fig. 1:**
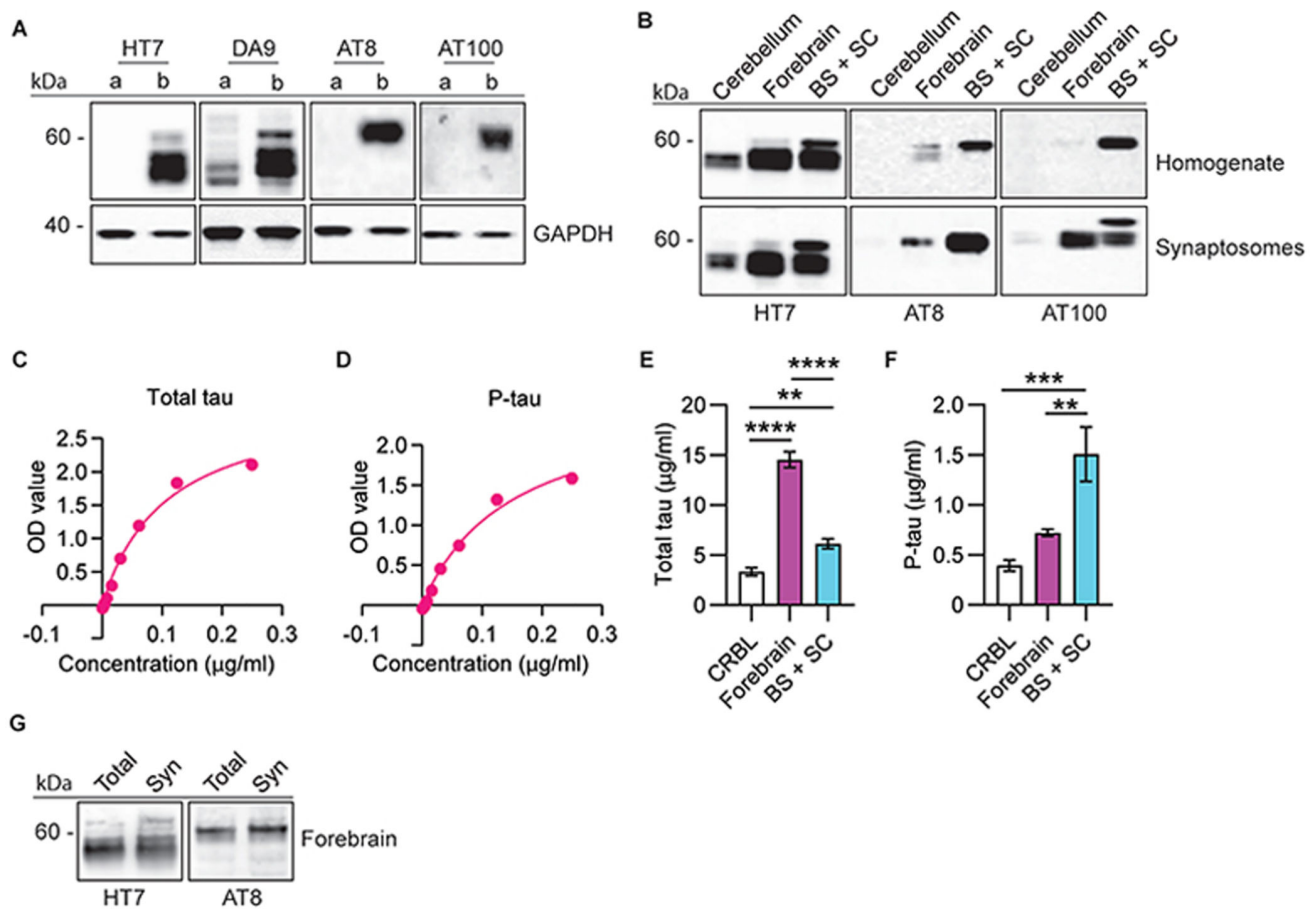
Biochemical characterisation of synaptic tau in TgP301S and Tg4510 mice. **(A)** Antibodies reactivity and specificity for human tau was evaluated in synaptosomes purified using sucrose gradients from pooled forebrains of control C57 black/6J mice (a) and TgP301S mice (b). HT7, AT8 and AT100 antibodies recognised only human tau whereas DA9 recognised both endogenous murine tau and the transgenic human tau isoforms. Notably, a higher molecular weight form of tau of about 64 kDa was seen with all antibodies, but it was the only band recognised by the specific human phospho-tau antibodies AT8 and AT100. **(B)** Tissue distribution of total (HT7-positive) and phosphorylated (AT8- and AT100-positive) tau was compared in total homogenates and synaptosomes from different CNS regions, including cerebellum, forebrain, and brain stem and spinal cord (BS + SC) of TgP301S mice. Synaptosomal tissue distribution replicates what was previously shown for total homogenates. **(C and D)** Standard curves for quantification of total (DA9-positive) and phosphorylated (AT8-positive) tau by ELISA. **(E and F)** Differential tissue distribution of total (HT7-positive) and phosphorylated (AT8-positive) tau in TgP301S synaptosomes quantified by ELISA. The results are expressed as means ± SEM (n=3). **p<0.01, ***p<0.001, ****p<0.0001 (one-way ANOVA Dunnett’s post hoc test). **(G)** Similar expression of total (HT7-positive) and phosphorylated (AT8-positive) tau was also found in Tg4510 homogenates (Total) and synaptosomes (Syn) obtained from Tg4510 mice forebrains.

**Fig. 2:**
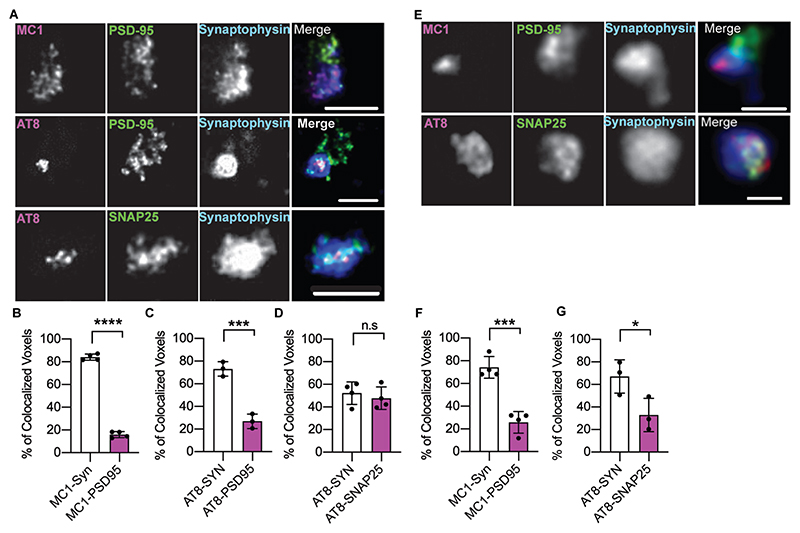
Tau detection by STED in synaptosomes purified from tau transgenic mouse and human Alzheimer’s disease brains. (**A**) Synaptosomes purified from TgP301S tau transgenic mice were immunolabeled with pre- and post-synaptic markers (presynaptic compartment, synaptophysin and SNAP25; postsynaptic compartment, PSD95) in addition to phosphorylated (AT8) or conformational (MC1) tau specific antibodies. Super resolution images show preferential colocalization of AT8-positive and MC1-positive tau with the presynaptic markers. (**B-D**) quantification of co-localisation of labelling with the different antibodies. (**E-G**) Same co-localisation experiments were performed in isolated human Alzheimer’s disease brain synaptosomes. Data represent the mean ± SEM. *p<0.05, ***p<0.001 (Independent t test). Scale bars: 1 μm in (**A**) and 0.5 μm in (**E**).

Synaptosomes from TgP301S and Tg4510 mice, as well as human AD brains, were characterised for the presence of pathological forms of tau (Fig. 1 and Suppl. Fig. 1-3). In all three preparations we confirmed the presence in synaptosomes of hyperphosphorylated tau recognised by the AT8 antibody (Fig. 1; Suppl. Fig. 1 and 2). AT100 antibodies recognised both TgP301S and human AD synaptosomal tau (Tg4510 was not tested) (Fig. 1 and Suppl. Fig. 3). Synaptosomal tau was mostly in an aggregated form, as evidenced by the presence of high molecular weight smears on gels and the enrichment of hyperphosphorylated tau in the Triton X100- and sarkosyl-insoluble fractions in both rodent and human samples (Suppl. Fig. 2 and 3). The enrichment of pathological tau in synaptosomes and its presence within the presynaptic compartment were also confirmed morphologically using high-resolution STED imaging (Fig. 2). Once confirmed that the synaptosomes from mouse and human preparations contained aggregated and hyperphosphorylated tau, we focused on its release mechanisms.

**Fig. 3:**
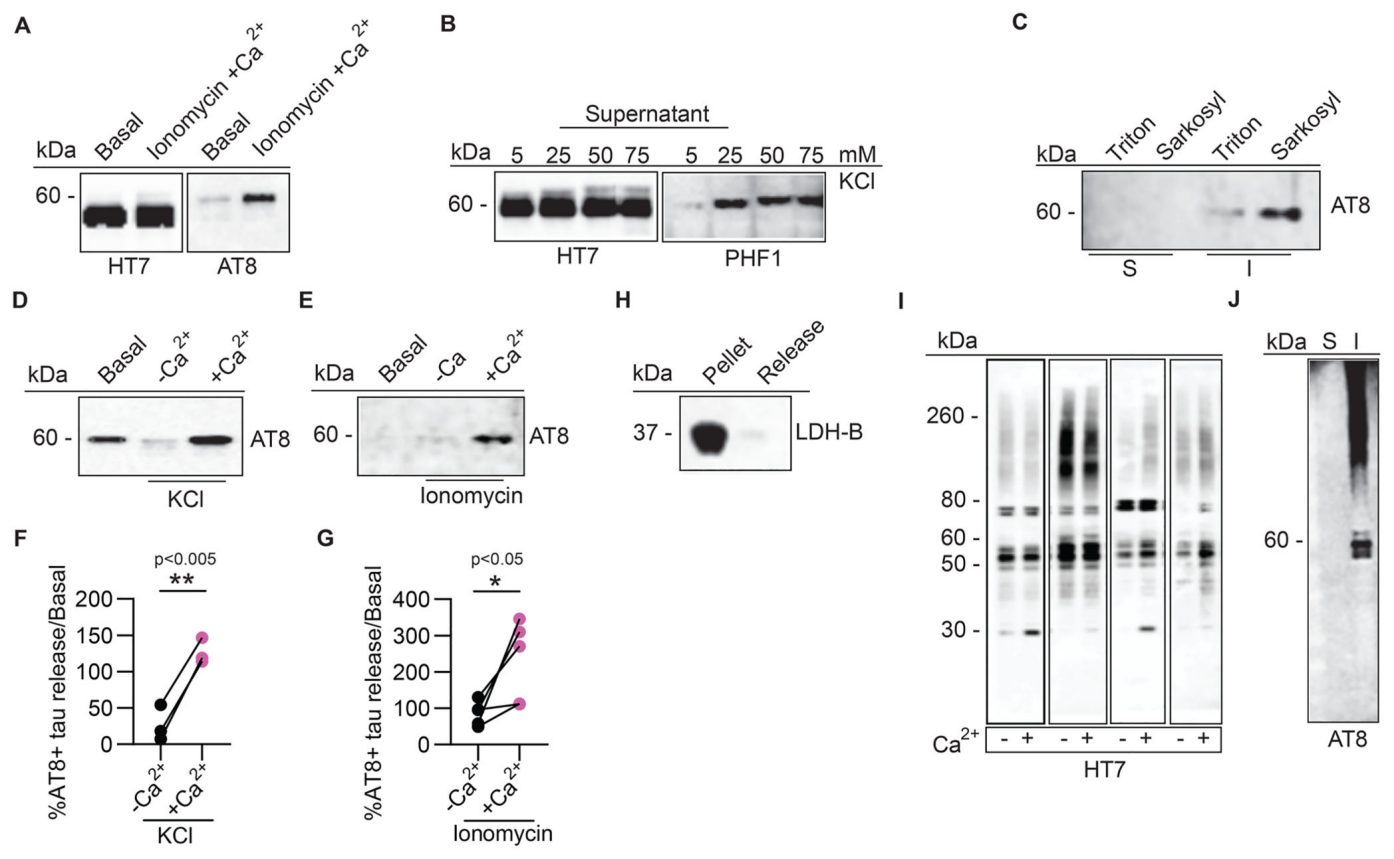
Tau release from TgP301S and human Alzheimer’s disease synaptosomes. **(A-G)** Representative immunoblots and quantification of tau release from synaptosomes isolated from TgP301S brains. Synaptosomes were either depolarized with 50 mM KCl or stimulated with 100 mM of the calcium ionophore ionomycin for 30 min at 37°C, as indicated. Both ionomycin **(A)** and KCl **(B)** caused the release of an AT8-positive and PHF1-positive tau, which was also detergent insoluble **(C)**. Both KCl- **(D)** and ionomycin- **(E)** induced release were dependent on the presence of extracellular calcium. Quantification of multiple experiments as in **(D)** and **(E)** is plotted in **(F)** and **(G)**. Two-tailed *t*-test with Welch’s correction t (3.611)=5.696, p=0.0064, N=3 independent experiments and t (4.698)=2.789, p=0.0416, N=5 independent experiments, for **(F)** and **(G)**, respectively. **(H)** LDH-B was not released after addition of ionomycin, confirming synaptosome integrity under these experimental conditions. **(I)** Synaptosomes from four independent samples of post-mortem human Alzheimer’s disease brains (Braak stage VI) were stimulated with 100 nM ionomycin with or without extracellular calcium and the supernatants analysed for HT7-positive tau. The arrowhead points to a 30 kDa tau fragment preferentially released in the presence of calcium. **(J)** AT8-positive tau was found in the insoluble (I**)** fraction of sarkosyl precipitated supernatants from Alzheimer’s disease brains.

Both ionomycin (Fig. 3A) and KCl (Fig. 3B) induced the release of high molecular weight, HT7-positive species of tau from TgP301S synaptosomes, which were also selectively recognised by both AT8 (Fig. 3A) and PHF1 (Fig. 3B) antibodies. Released AT8-positive tau was mainly found in the detergent-insoluble fractions (Fig. 3C), thus confirming that in these transgenic mice, tau residing within nerve terminals and released following depolarization exists in a pathologically hyper-phosphorylated, aggregated form.

**Fig. 4:**
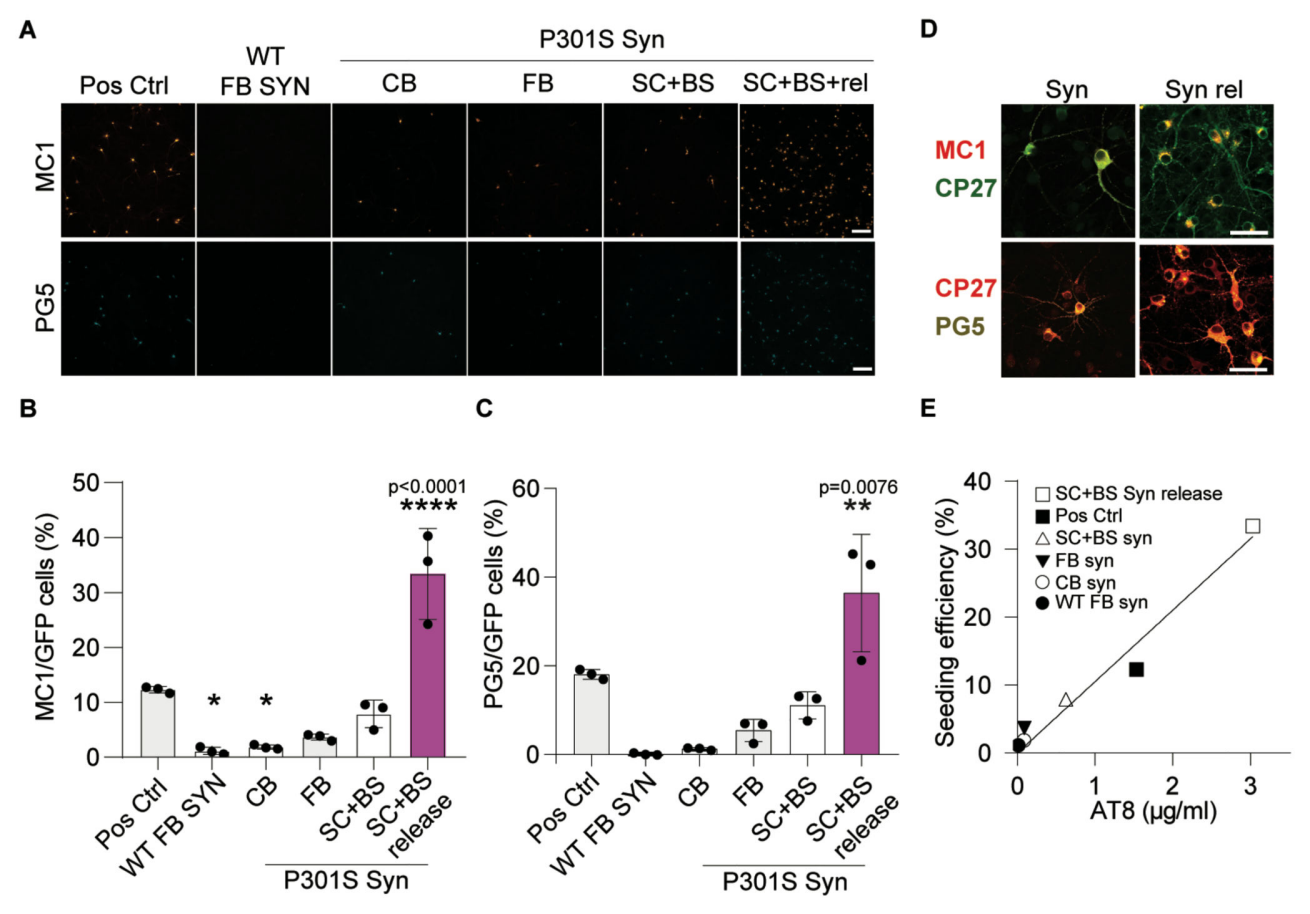
TgP301S synaptosomes and insoluble tau released from them are seeding competent. **(A)** Representative images of rat cortical neurons expressing GFP and human P301S tau treated for 7 days with synaptosome extracts from different CNS regions (SC+BS = spinal cord + brain stem, FB = forebrain, CB = cerebellum), or the insoluble synaptosome release fraction from SC+BS (SC+BS release), from WT or TgP301S mouse brain tissues. Neurons were immunostained with either MC1 or PG5 antibodies. Positive control (Pos Ctrl) is described in Material and Methods. Scale bar **=** 100 µm. (**B and C)** Quantitation of seeding efficiency measured as percent of MC1 **(B)**, or PG5 **(C)** positive cells to total transduced cells. Data presented as mean ± SEM of n=3, and as individual points; one-way ANOVA followed by Tukey’s multiple comparison’s test) F (5, 12) =35.15, p<0.0001 and F (5, 12) =17.49, p<0.0001 for MC1 and PG5, respectively. (**D)** High magnification images of MC1 or PG5 and CP27 stained neurons. Scale bar = 50 µm. **(E)** Correlation of seeding efficiency versus AT8-positive tau concentration in tested samples.

To confirm the calcium dependency of tau release, we repeated the above experiments in the presence or absence of extracellular calcium (Fig. 3D-G). Both KCl- (Fig. 3D and F) and ionomycin-mediated (Fig. 3E and G) release of AT8-positive tau was significantly reduced in the absence of extracellular calcium. Qualitatively similar results were obtained in synaptosomes from Tg4510 mice (data not shown). The release of AT8-positive and PHF1-positive tau was not the consequence of ionomycin-induced synaptosome damage, as demonstrated by the absence of release of lactate dehydrogenase (LDH) in the supernatant (Fig. 3H). Interestingly, both synaptosomal and released tau obtained from TgP301S brains were seeding competent as shown in experiments performed using transfected rat cortical neurons as recipient cells (Fig. 4).

Tau release was more difficult to measure in human synaptosomes, possibly due to their fragility and/or because of the presence of lower levels of pathological tau, compared with human mutant tau-overexpressing transgenic mice. Supernatants of ionomycin-stimulated human synaptosomes isolated from frontal cortex of four post-mortem Alzheimer’s disease patients contained significant amounts of HT7-positive tau. Among HT7 immunoreactive species, whose release was clearly calcium-dependent, a low molecular weight band of about 30 kDa was noticeable (Fig. 3I, arrowhead). This fragment is possibly related to the truncated form of tau previously described in human synaptosomes (Allen et al., 2002).

When we looked for AT8-positive hyper-phosphorylated tau in the supernatants of these Alzheimer’s disease samples, no detectable signal was initially found (not shown). Considering the possibility that only small amounts of AT8-positive tau are released from human Alzheimer’s disease synaptosomes, supernatants were treated with 1% sarkosyl to concentrate any aggregated tau released upon stimulation. Using this protocol, a strong AT8-positive signal was observed in the sarkosyl-insoluble fraction of the supernatants (Fig. 3J).

This finding is important for two reasons: first, it confirms that the initial inability to detect hyper-phosphorylated tau in the supernatants from stimulated human synaptosomes was likely due to its low levels; second, and most relevant, it demonstrates that hyper-phosphorylated and aggregated tau is released from human Alzheimer’s disease synaptosomes stimulated by treatment with calcium ionophores, such as ionomycin. Based on these results, for subsequent release experiments from human synaptosomes, we used sarkosyl precipitation of the supernatant as a standard procedure to concentrate human pathological tau.

### Tau release requires SNAP25 integrity

**Fig. 5:**
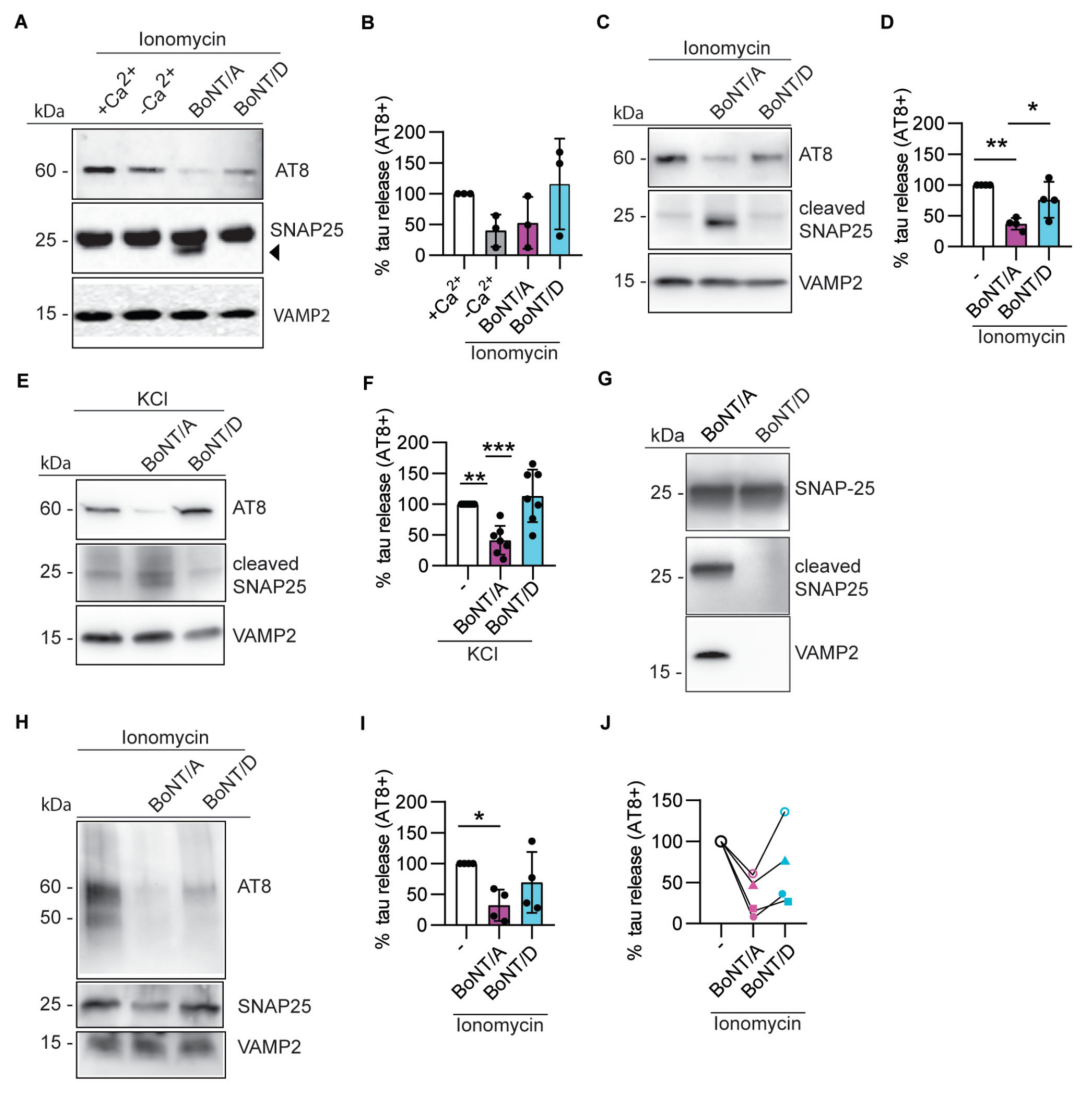
Cleavage of SNAP25 by BoNT/A decreases tau release from TgP301S, Tg4510 and human Alzheimer’s disease synaptosomes. (**A and B**) Representative immunoblots of AT8-positive tau released from synaptosomes isolated from TgP301S brains and stimulated with 100 nM ionomycin in the presence or absence of BoNT/A or BoNT/D **(A**), and its quantification from multiple experiments **(B)**. The arrowhead shows the position of cleaved SNAP25. One-way ANOVA F (3,8) =2.029, p=0.1885. N=3 independent experiments. (**C and D)** Similar experiments as in **(A, B)** using synaptosomes isolated from Tg4510 brains. One-way ANOVA F (2, 6) =12.79, p=0.0069); Tukey’s multiple comparisons test **p=0.0074, *p=0.0201; n=4 independent experiments. (**E and F)** Similar experiments as in **(A, B)** using TgP301S synaptosomes stimulated with KCl. One-way ANOVA F (2,18) =13.05, p=0.0003); Tukey’s multiple comparison test **p=0.0028, ***p=0.0004; n=7 independent experiments. (**G)** Antibodies directed against full length SNAP25, cleaved SNAP25, and full length VAMP2 were used to monitor the cleavage efficiency of BoNT/A and D. (**H-J)** Representative immunoblots of AT8-positive tau released from synaptosomes isolated from the frontal cortex of Alzheimer’s disease patients (Braak stage VI) and stimulated with 100 nM ionomycin **(H)**. The quantification of the data in **H** is shown in **I** and **J. J** highlights the effects on individual patient’s synaptosomes. One-way ANOVA (F (2,9) =4.449, p=0.0453); Tukey’s multiple comparison test *p=0.0374; n=4. In all cases, synaptosomes were pre-incubated for 30 min at 37°C with either BoNT/A or BoNT/D and then stimulated for 30 min at 37°C with either 100 nM ionomycin or 50 mM KCl. The released material was precipitated with sarkosyl before being loaded on the gel as described in the text.

The observed calcium dependency of aggregated and seeding-competent tau release suggests, but does not prove, that regulated, vesicular secretion at synapses plays a functional role in this process. To ascertain whether the secretory machinery is implicated in pathological tau release, we took advantage of the specificity of botulinum neurotoxins to prevent exocytosis by cleaving specific SNARE proteins (Schiavo et al., 2000; Pirazzini et al., 2017). We pre-incubated synaptosomes from TgP301S and Tg4510 mice, as well as human Alzheimer’s disease synaptosomes, with botulinum neurotoxins A and D (BoNT/A and BoNT/D), either in combination or individually. BoNT/D is known to cleave the synaptic vesicle protein VAMP/synaptobrevin, whereas BoNT/A specifically cuts SNAP25, a SNARE protein found at pre-synaptic and extra-synaptic sites (Schiavo et al., 2000; Pirazzini et al., 2017). VAMP/synaptobrevin and SNAP25 form the pre-synaptic SNARE complex with syntaxin1/2, which is required for the fusion of synaptic vesicles with the neuronal membrane and the release of neurotransmitters (Sudhof and Rothman, 2009). We found that when used in combination, the two neurotoxins significantly inhibited ionomycin-induced tau release in synaptosomes from TgP301S mice (data not shown). Interestingly, the inhibitory activity seemed to be predominantly mediated by BoNT/A and to a lesser extent by BoNT/D (Fig. 5A and B). Importantly, this result was confirmed in synaptosomes from Tg4510 mice (Fig. 5C and D). In Tg4510 synaptosomes, we found that BoNT/A was able to block tau release evoked not only by ionomycin, but also KCl (Fig. 5E and F). By utilising an antibody detecting full length SNAP25 (Fig. 5A and G) or a polyclonal antibody that recognizes only cleaved SNAP25 (Antonucci et al., 2008) (Fig. 5C, E, G), we confirmed that SNAP25 cleavage parallels the inhibition of tau release by BoNT/A. In spite of the lack of significant effects on tau release, BoNT/D was also fully active, as demonstrated by the cleavage of VAMP/synaptobrevin when using an antibody that recognises the full-length form of the protein (Fig. 5A, C, E, G). Importantly, the selective inhibition of tau release by cleavage of SNAP25 by BoNT/A was confirmed also in human Alzheimer’s disease brain synaptosomes (Fig. 5H-J).

### Activation of mGlu2/3 receptors inhibits pathological tau release and propagation

An important synaptic connection affected very early in Alzheimer’s disease pathology is the entorhinal-dentate gyrus (EC>DG) pathway. This glutamatergic pathway is modulated by presynaptic glutamate auto-receptors (Witkin et al., 2017). We therefore decided to investigate whether these auto-receptors could also play a role in tau release.

**Fig. 6:**
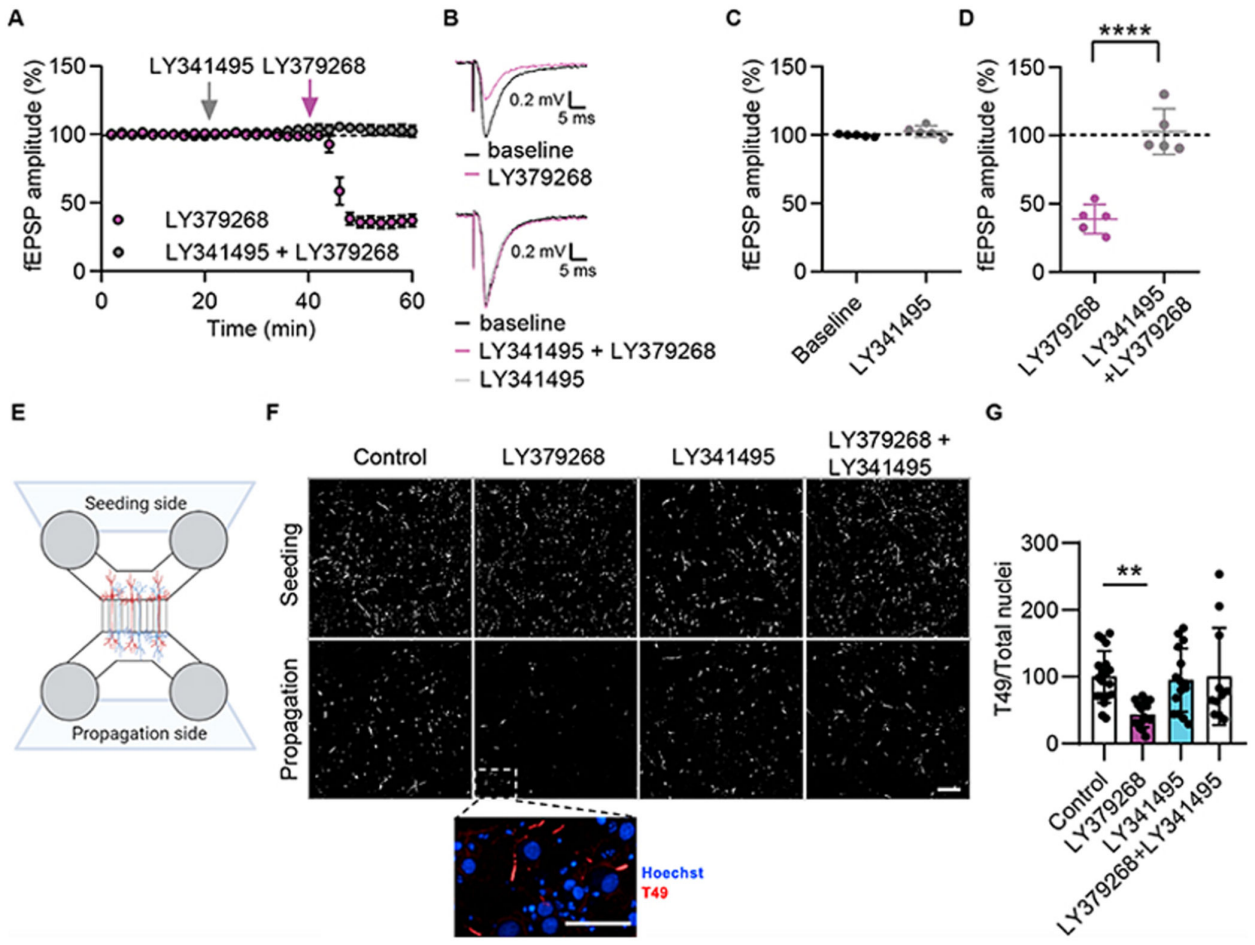
mGlu2/3 modulates synaptic transmission in brain slices and tau propagation in cultured rat neurons. **(A)** The mGlu2/3 agonist LY379268 depressed glutamatergic synaptic transmission (fEPSP amplitude) in dentate gyrus *ex-vivo* slices (purple circles, N=5). Pre-incubation with the mGlu2/3 antagonist LY341495 completely prevented the LY379268 inhibitory effect (grey circles, N=5). Each point represents the average of four sweeps and the mean ± SEM. (**B)** Example traces, after 20 minutes of agonist, or agonist plus antagonist, addition, from one experiment included in **(A)**. (**C and D)** Quantification of drugs effects at the end of the 20 perfusions. The antagonist by itself did not change fEPSP amplitudes **(C)**, but completely prevented the agonist effect **(D)** (unpaired Student’s t test (****P<0.0001)). (**E)** Schematic representation of the microfluidic device used to study tau propagation *in vitro*. (**F)** Representative images of release and propagation of seeded human Alzheimer’s disease tau in DIV21 rat primary cortical culture seeded at DIV7 and stained with the rodent-specific T49 tau antibody (AlexaFluor647) and Hoechst in both the seeding (top row) and propagation chambers (bottom row). The propagation chamber was treated with 10 µM LY379268, 10 µM LY341495, or both combined. No effect on aggregation was observed when the drugs were added to the seeding chamber. Scale bars, 50 μm. (**G)** Quantification of the mGlu 2/3 drugs effects when added to the propagation chamber. One-way ANOVA F (3, 57) =5.826, p=0.0015; Dunnett’s multiple comparison test vs control p=0.0016. N=3 independent experiments.

By performing electrophysiological experiments in rat brain slices, we first confirmed that glutamatergic transmission in the EC>DG pathway is potently inhibited by LY379268, a well characterized agonist of the metabotropic glutamate receptor 2/3 (mGlu2/3) (Bond et al., 2000; Monn et al., 1999; Sanger et al., 2013) (Fig. 6A-D). We also showed that this inhibitory effect on synaptic transmission was prevented by the mGlu2/3 antagonist LY341495 (Johnson et al., 1999) (Fig. 6A-D).

Confident that we identified appropriate tool compounds for modulating synaptic transmission in Alzheimer’s disease-relevant synapses, we then moved to a simplified *in vitro* cellular system. As we previously demonstrated, microfluidic chambers can be effectively used to quantify tau release and propagation, and to test the effects of compounds that can modulate this process (Katsikoudi et al., 2020). Therefore, we decided to test whether mGlu2/3 modulators could affect tau release and propagation in this system.

Rat cortical neurons were plated in microfluidic devices (design depicted in Fig. 6E) and seeded with human pathological tau at DIV7. Cultures were then treated in the propagation compartment (Fig. 6E) with 10 µM LY379268 and LY341495, alone or in combination. Neurons were fixed at DIV21, stained, and analysed for aggregated tau using the rodent-specific tau T49 antibody. As shown in Fig. 6F and G, LY379268 significantly decreased tau propagation when compared to vehicle control. Importantly, although the mGlu2/3 antagonist LY341495 had no effects on its own, it completely prevented the inhibitory effects of the agonist LY379268 on pathological tau release and propagation (Fig. 6F, G).

**Fig. 7:**
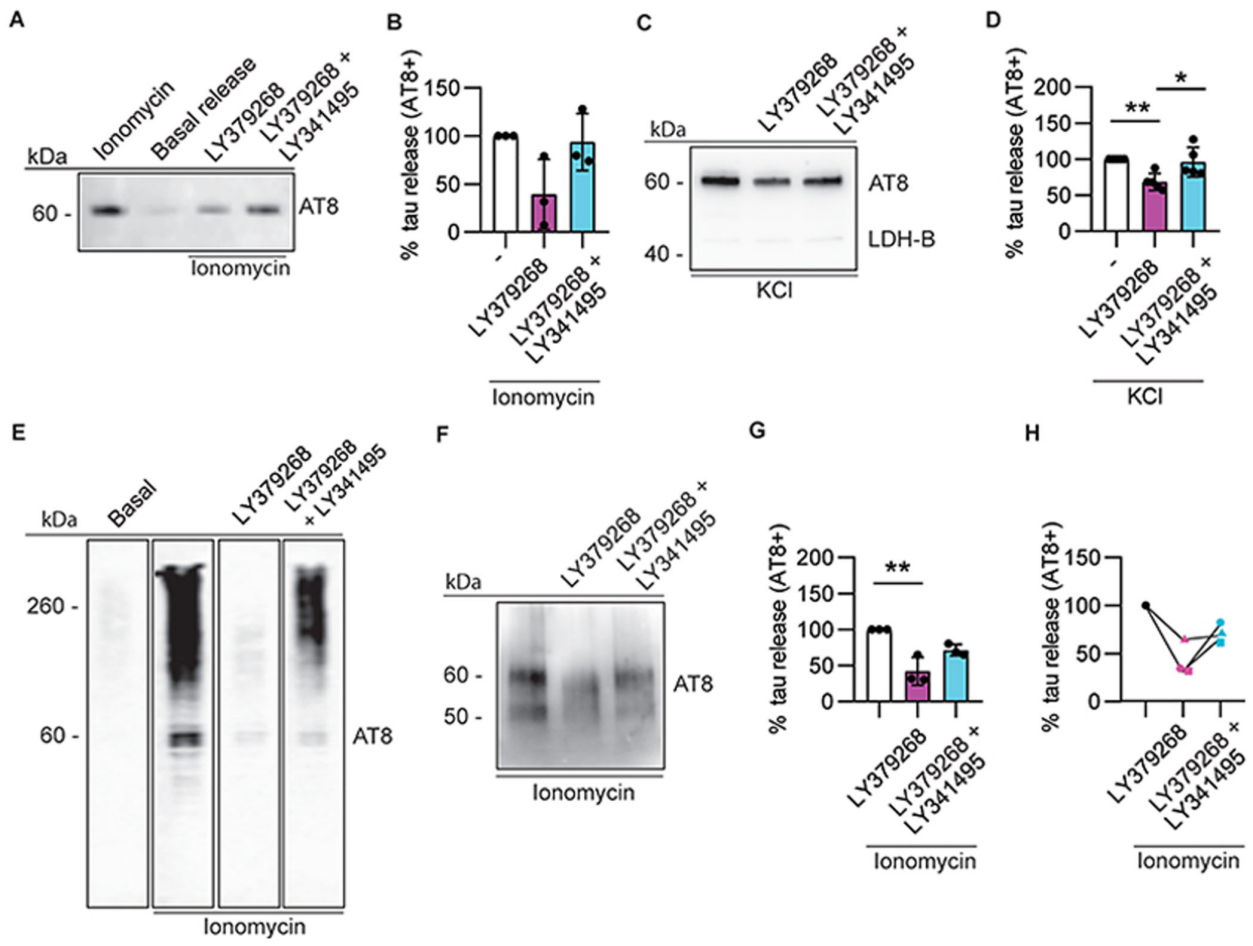
mGlu2/3 receptor activation prevents tau release from TgP301S, Tg4510 and human Alzheimer’s disease synaptosomes. **(A and B)** Representative immunoblots of AT8-positive tau released from synaptosomes isolated from TgP301S and stimulated with 100 nM ionomycin in the presence of mGlu2/3 modulators **(A)** and its quantification **(B)**. One-way ANOVA F (2,6) =2.179, p=0.1943. (**C and D)** Representative immunoblots of AT8-positive tau released from synaptosomes isolated from TgP301S and stimulated with 50 mM KCl in the presence of mGLu2/3 modulators **(C)** and its quantification **(D)**. One-way ANOVA (F (2, 12) = 7.965, p=0.0063), Tukey’s multiple comparison test **p=0.0085 (LY379268), *p=0.0190 (LY379268 + LY341495), N=5 independent experiments. (**E**) Sarkosyl-insoluble AT8-positive tau is released by 100 nM ionomycin in a representative sample of human Alzheimer’s disease synaptosomes, with very little being released under basal conditions. LY379268 blocked tau release upon treatment with ionomycin and this effect was prevented by LY341495. (**F-H)** as in **(E)** but using an independent group of Braak stage VI Alzheimer’s disease patients’ synaptosomes. The quantification of the data in **F** is shown in **G** and **H. H** highlights the effects on individual patient’s synaptosomes. One-way ANOVA (F (2,6) =17.39, p=0.0032), Tukey’s multiple comparison test **p=0.0026 (LY379268).

Since the mGlu2/3 agonist LY379268 was able to block synaptic transmission in rodent brain slices, as well as tau propagation in rodent primary neuron cultures, we then tested its ability to modulate tau release from brain synaptosomes. LY379268 inhibited ionomycin- and KCl-induced AT8-positive tau release in TgP301S (Fig. 7A-D), Tg4510 (not shown) and human Alzheimer’s disease synaptosomes (Fig. 7E-H). This inhibitory effect was significant irrespective of whether ionomycin (Fig. 7A, B) or KCl (Fig. 7C, D) were used as a stimulus. As shown in the propagation assay displayed in Fig. 6, the block of pathological tau release caused by LY379268 was prevented by the mGlu2/3 antagonist LY341495 in both TgP301S and human Alzheimer’s disease synaptosomes (Fig. 7A-H).

## Discussion

Understanding the mechanisms of pathological tau spreading in Alzheimer’s disease brains has become a focus of recent Alzheimer’s disease research due to the emerging correlation between tau spreading and cognitive decline during disease progression in these patients. Until recently, the extent of tau spreading could only be determined post-mortem (Braak and Braak, 1991; Spillantini and Goedert, 2013; Braak and Del Tredici, 2016; Wang and Mandelkow, 2016; Goedert et al., 2017); however, recent advances have enabled the possibility to follow this process in living patients utilising newly developed tau PET tracers (Johnson et al., 2016; Scholl et al., 2016; Franzmeier et al., 2019; Vogels et al., 2020).

Tau pathology “spreading” in the brain could be the result of a time-dependent vulnerability of adjacent neurons. However, an increasing body of *in vitro* and *in vivo* evidence suggests that active transfer of pathological tau can occur between synaptically-connected neurons (Clavaguera et al., 2009; de Calignon et al., 2012; Liu et al., 2012; Ahmed et al., 2014; Lewis and Dickson, 2016; Wang and Mandelkow, 2016). One critical step in validating the synaptic transfer hypothesis is to study native synapses and demonstrate both the presence and the release of pathological tau from presynaptic terminals. Focusing on rodent studies, Kremer and colleagues analysed synaptosomes purified from the P301L tauopathy mouse model (which expresses the same mutation as in Tg4510 mice, but under a different promoter) and found an enrichment of AT8-positive tau in hippocampal synaptosomes (Kremer et al., 2011). However, their focus was on the time-dependent changes of post-synaptic tau and its correlation to cognitive decline, rather than the mechanism of release. More recently, both Yamada *et al*. (Yamada et al., 2014) and Wu *et al*. (Wu et al., 2015) demonstrated that normal tau, as well as over-expressed tau is released *in vivo* upon neuronal stimulation via pharmacological modulation and dialysis, or after optogenetic or chemogenetic stimulation. Limited insights were generated, however, on the release mechanism of pathological tau.

Focusing on human synaptosomes, Fein *et al*. (Fein et al., 2008) and Sokolow *et al*. (Sokolow et al., 2012) identified hyper-phosphorylated tau in FACS-sorted Alzheimer’s disease synaptosomes, particularly those isolated from the entorhinal cortex and hippocampus, which partially co-localised with amyloid beta (Aβ). These authors found that a low molecular weight, C-terminal truncated form of tau was preferentially released. Furthermore, Hyman and colleagues (Tai et al., 2014) have shown by both immunolocalization and immuno-electron microscopy that aggregated tau can be found in both pre- and post-synaptic compartments in human Alzheimer’s disease synaptosomes. Our STED results confirm and extend these findings.

Here, we demonstrate that pathological tau is present in synaptosomes isolated from the brains of two different rodent models overexpressing human mutant tau as well as from brains of patients with Alzheimer’s disease. Furthermore, we have demonstrated that pathological tau is released in a depolarisation and calcium-dependent manner by a mechanism involving specific SNARE proteins. Selective release from synaptosomes of higher molecular weight, hyper-phosphorylated, aggregated human tau was induced by depolarisation, or by direct calcium influx induced by the selective calcium ionophore ionomycin in all synaptosome preparations.

Calcium-dependent fusion of vesicular organelles localised at nerve terminals, including synaptic vesicles, large dense-core granules and secretory lysosomes, to name but a few, is mediated by an array of SNARE proteins, which form specific SNARE complexes enabling membrane fusion (Sudhof and Rothman, 2009). Several of these SNARE proteins are also irreversibly inactivated by treatment with different botulinum toxins. We have found that both KCl- and ionomycin-induced tau release can be prevented by incubating synaptosomes with BoNT/A, indicating that the SNAP25-dependent fusion of AT8-positive tau organelles within synaptic terminals could represent a crucial step in facilitating spreading of tau pathology throughout the brain. In our hands, BoNT/D, which specifically cleaves VAMP/synaptobrevin, was much less effective than BoNT/A in halting pathological tau release.

Our results are therefore different from those of Pooler *et al*. (Pooler et al., 2013), who studied the release of non-phosphorylated, physiological tau from rat cortical neurons in culture. In their study, the stimulated release of normal tau was sensitive to tetanus toxin which, similarly to BoNT/D, cleaves VAMP/synaptobrevin. A possible explanation of this discrepancy is that different mechanisms underlie the release of physiological versus pathological tau. Another possibility is that cultured rat neurons have a release machinery, which is slightly different from that of acutely dissociated mouse and human brain synaptosomes. More in-depth studies are now warranted to better define the biochemical and morphological properties of the synaptic compartments containing AT8-positive tau and the molecular machinery responsible for their exocytosis.

In the last decade, BoNTs have been approved for a variety of therapeutic applications, including for spasticity, migraine, and neuropathic pain (Tighe and Schiavo, 2013; Pirazzini et al., 2017). However, in the context of our present research, we consider BoNTs mostly as valuable biochemical tools to dissect the molecular machinery of synaptic tau release. That said, several endogenous neurotransmitters acting on pre-synaptic receptors (e.g., metabotropic serotonin or glutamate receptors) have been shown to functionally mimic the physiological effects of BoNTs and, more specifically, to induce a G-protein β/γ mediated inhibition of SNAP25 (Zhang et al., 2011; Upreti et al., 2013).

We focused here on presynaptic mGlu2/3 because of their exquisite localisation in Alzheimer’s disease-relevant brain areas and their established role in sequestering synaptic SNAP25. Using rat brain slices, we confirmed that the mGlu2/3 agonist LY379268 efficiently blocks synaptic transmission in the hippocampal dentate gyrus and this inhibition was prevented by the selective mGlu2/3 antagonist LY341495. When we re-created synaptic connections between cortical rat neurons in microfluidic devices, we showed that pathological tau release and propagation into postsynaptic cells was also blocked by LY379268, and this inhibitory effect was prevented by LY341495.

Pharmacology does not always translate from rodents to humans, and this is also the case for mGlu receptors (Sheahan et al., 2018). To start bridging this important functional gap, we compared the effects of the mGlu2/3 agonist in both mouse and human Alzheimer’s disease synaptosomes. LY379268 blocked tau release from synaptosomes isolated from both species, an effect blocked by LY341495. We believe this is the first report of a well characterised pharmacological agent to affect tau release in human Alzheimer’s disease brain tissue. Supporting a role for these mechanisms *in vivo*, Yamada *et al*. (Yamada et al., 2014) found by *in vivo* brain micro-dialysis that LY341495 potentiated total tau release. This was attributed to the block of a tonic activation of presynaptic mGlu receptors by endogenous glutamate. Future work with selective mGlu2 versus mGlu3 receptor ligands will help addressing which subtype is more relevant to the effects described here. Likewise, more studies will be required to formally prove that these effects are mediated by SNAP25 inhibition, as suggested in the literature (Zhang et al., 2011; Upreti et al., 2013).

Although we demonstrated calcium- and SNARE-dependent pathological tau release from cortical synaptosomes from both mouse tauopathy models and post-mortem human Alzheimer’s disease brains, it is likely that other mechanisms of pathological tau release and propagation occur under different conditions. For example, tau release could depend on the synaptic tau load which would be, in turn, related to disease progression. This could explain why we detected higher tau release in Tg4510 forebrains than in TgP301S brains, with the former being less dependent on extracellular calcium (data not shown). Recently, it was suggested that results obtained using the Tg4510 mice should be interpreted with caution as transgene insertion alters the expression of mouse genes and might thus affect neurodegeneration rather than overexpression of human mutant tau itself (Gamache et al., 2019). However, here we have shown that tau release from synaptosomes, its dependency on SNARE proteins and modulation by mGluRs are replicated in two different tau transgenic models as well as Alzheimer’s disease patient brains, suggesting that the basic machinery of pathological tau release is highly conserved across disease models and species.

The interplay with Aβ fibrils, as well as the neuroinflammation status of the specific brain area (Asai et al., 2015) may also contribute to the diversity of the secretory and propagation mechanisms involved in different stages of Alzheimer’s disease. Despite this possible underlying heterogeneity, our mechanistic findings suggest that the release machinery of pathological tau from rodent and human synapses is conserved and involves the calcium-dependent fusion of synaptic organelles with the pre-synaptic plasma membrane. A better understanding of the involvement of SNARE proteins in pathological tau release could lead to clinical exploration of pharmacological approaches targeting these specific presynaptic mechanisms, leading to the development of therapeutic strategies blocking tau spreading and disease progression.

## Supplementary Material

Supplementary Material

## Significance statement

Pathological tau release and propagation is a key neuropathological event underlying cognitive decline in Alzheimer’s disease patients. In this paper, we describe the role of regulated exocytosis, and the SNARE protein SNAP25, in mediating tau release from rodent and human synaptosomes. We also show that a selective mGluR2/3 agonist is highly effective in blocking tau release from synaptosomes and tau propagation between neurons, opening the way to the discovery of novel therapeutic approaches to this devastating disease.

## Abbreviations

AAV2: adeno-associated virus 2
aCSF: artificial Cerebrospinal Fluid
Aβ: amyloid beta
BME: β-mercaptoethanol
BS: Brain Stem
BSA: Bovine Serum Albumin
BoNT/A and D: Botulinum Neurotoxin serotype A and D
CB: Cerebellum
DG: Dentate Gyrus
DIV: Days In Vitro
EC: Entorhinal Cortex
fEPSP: field Excitatory Postsynaptic Potential
GFAP: Glial Fibrillary Acidic Protein
GFP: Green Fluorescent Protein
HRP: Horseradish Peroxidase
Iba1: Ionized calcium binding adaptor molecule 1
mGlu 2/3: metabotropic Glutamate receptor 2/3
MPP: medial perforant path
NR2B: N-Methyl D-aspartate receptor subtype 2B
PET: Positron Emission Tomography
PP: Paired Pulse
RCN: Rat Cortical Neuron
SC: Spinal Cord
SNAP25: Synaptosome Associated Protein of 25 kDa
SNARE: Soluble N-ethylmaleimide sensitive factor attachment protein complex
