# Supplementary material for: Metabotropic glutamate receptors modulate exocytotic tau release and propagation
Source: J Pharmacol Exp Ther. Author manuscript; Available in PMC 2026 May 28. (PMC7619109; doi:10.1124/jpet.122.001307)
Supplement: Supplementary Material [file EMS213898-supplement-Supplementary_Material.pdf]

SNAP25 is essential for pathological tau release and propagation

Francesca Mazzo, Ioana Butnaru, Olivera Grubisha, Elena Ficulle, Helen Sanger, Griffin Fitzgerald, Feng Pan, Francesca Pasqui, Tracey Murray, James Monn, Xia Li, Michael Hutton, Suchira Bose, Giampietro Schiavo and Emanuele Sher

Journal of Pharmacology and Experimental Therapeutics

JPET-AR-2022-001307

Supplementary Figure and Legends:

Supplementary Fig. 1

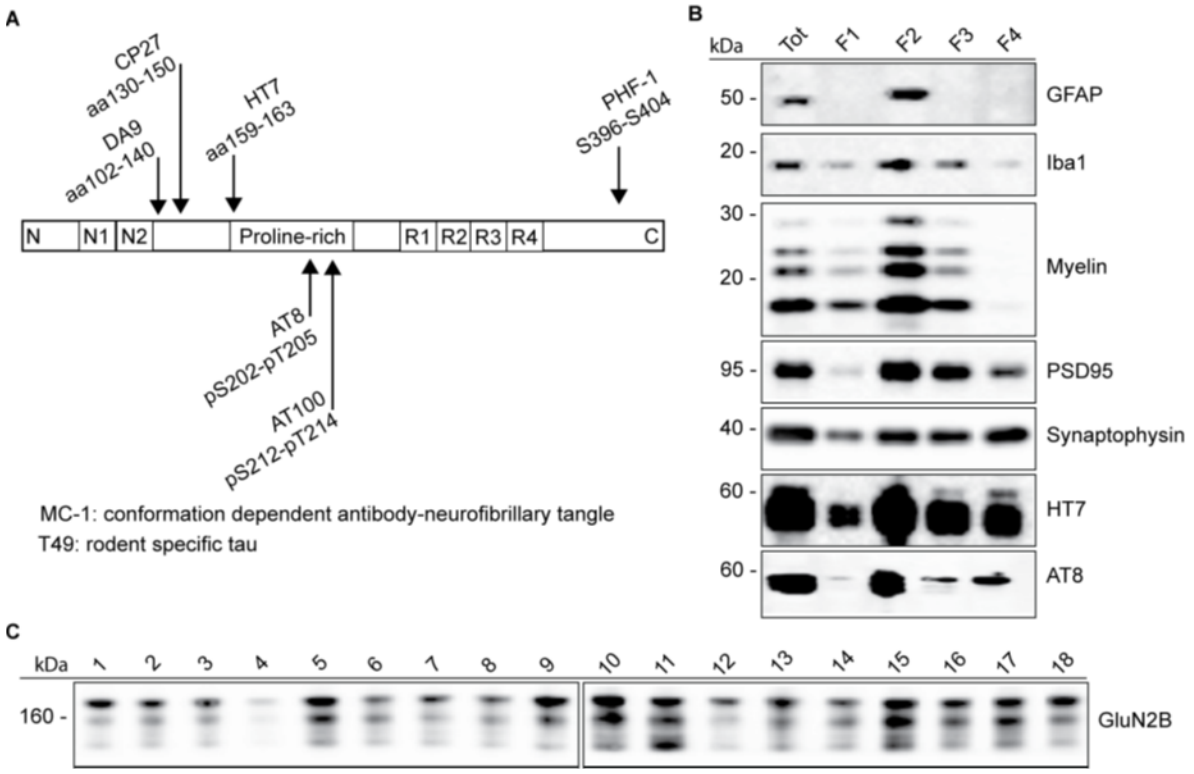

Supplementary Fig. 1. Characterisation of TgP301S and human Alzheimer's disease synaptosomes.

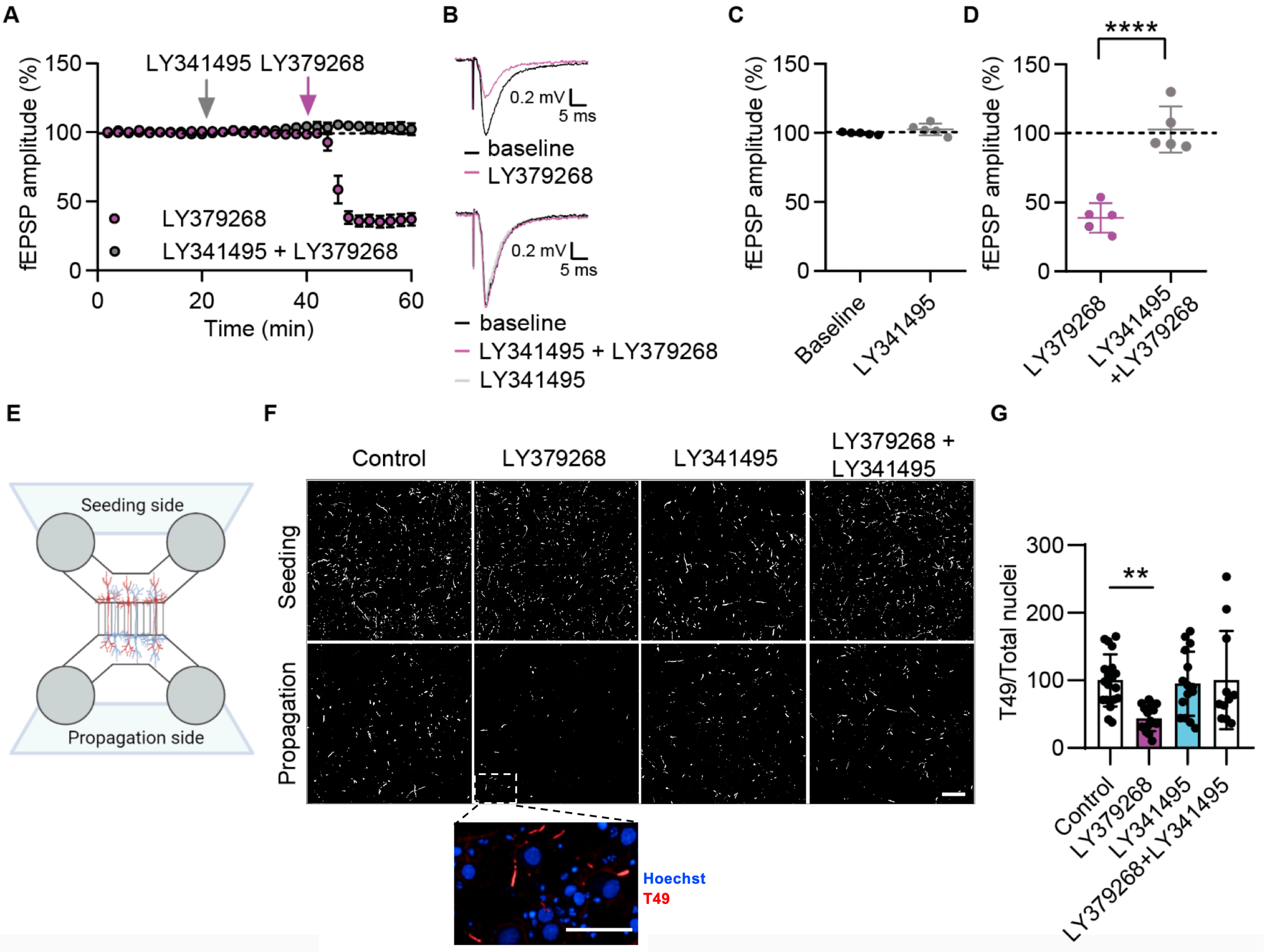
A
fEPSP amplitude (%)
150
100
50
0
LY341495 LY379268
LY379268
LY341495 + LY379268
0
20
40
60
Time (min)
B
0.2 mV
5 ms
baseline
LY379268
0.2 mV
5 ms
baseline
LY341495 + LY379268
LY341495
C
fEPSP amplitude (%)
Baseline
LY341495
D
****
fEPSP amplitude (%)
LY379268
LY341495 +LY379268
E
Seeding side
Propagation side
F
Control
LY379268
LY341495
LY379268 + LY341495
Seeding
Propagation
Hoechst
T49
G
T49/Total nuclei
300
200
100
0
**
Control
LY379268
LY341495
LY379268+LY341495

(A) A schematic representation of the 441 amino acid isoforms of the human tau protein and the defined epitopes recognized by each tau antibody. HT7 and DA9 are phosphorylation-independent antibodies. AT8 and AT100 are phosphorylation-dependent antibodies. MC1 is a conformational-dependent antibody. C, carboxy-terminal; N, amino-terminal. N1-2, two inserts of 29 amino acids at the N-terminal part (exon 2 and 3); R1-4, microtubule-binding repeats. **(B)** Pooled forebrains from TgP301S mice (21 weeks) were homogenised and the S1 fraction (TOT) was applied onto a gradient of Percoll, and four fractions obtained. The same amount of protein for each fraction was loaded on gels as described in Materials and Methods. The enrichment of synaptic proteins (PSD95 and synaptophysin), contaminants from astroglia (GFAP), microglia and macrophages (Iba1) and myelin were analysed, along with the presence of total (HT7-positive) and pS202/pT205 tau (AT8-positive). **(C)** Synaptosomes purified with a sucrose gradient from normal and AD frontal cortex and hippocampus were analysed for GluN2B degradation, a validated marker of synaptosomal integrity (Bayes et al., 2014). The samples show three main bands: the top band corresponding to the full-length protein, and the lower bands corresponding to degraded GluN2B. The ratio of intensities of the top band over the second band provides the HUman Synapse Proteome Integrity Ratio (HUSPIR) ratio (Bayes et al., 2014). The antibody used was designed against the C-terminal region of GluN2B.

Supplementary Fig. 2

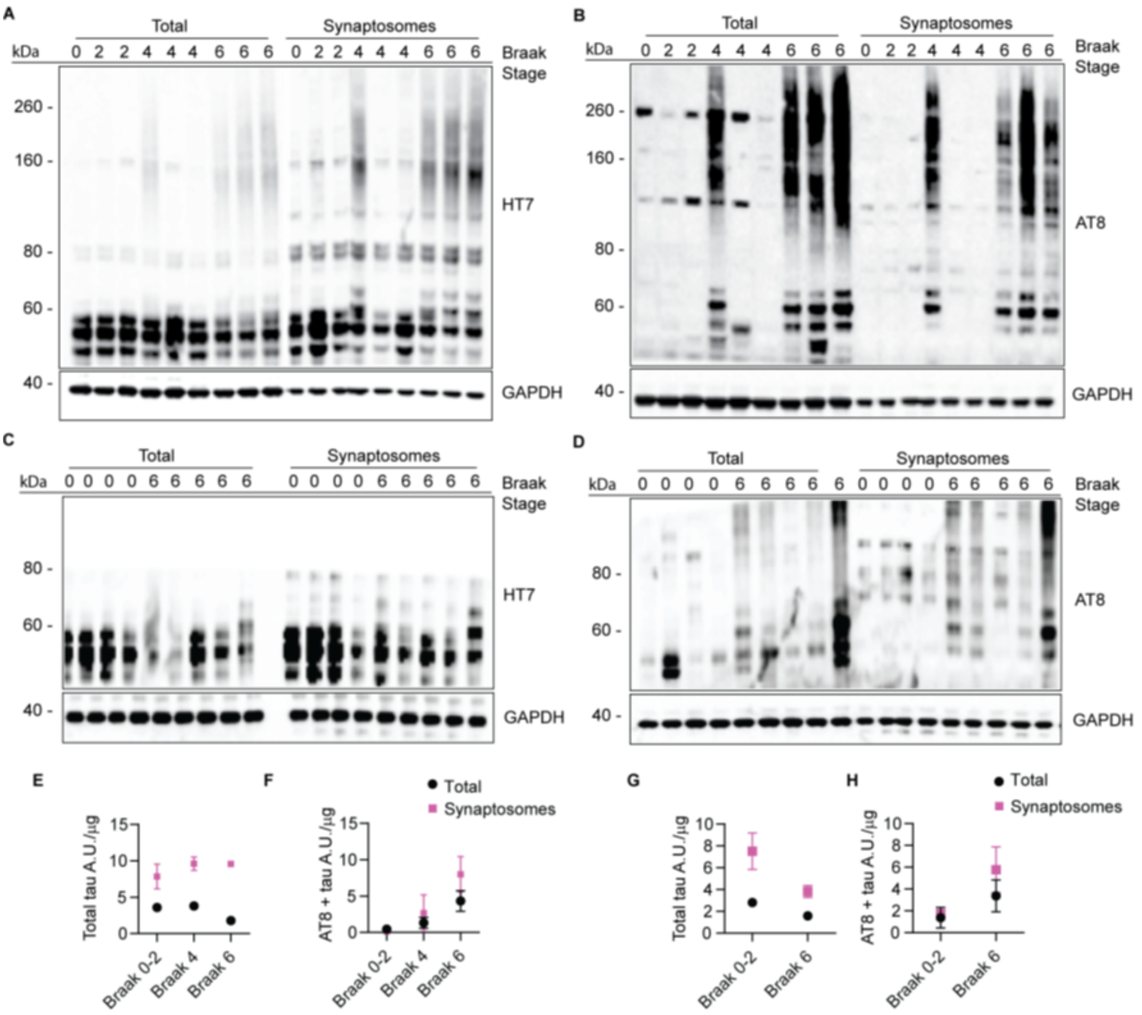

Supplementary Fig. 2. Expression of hyper-phosphorylated tau in human Alzheimer's disease synaptosomes.

(A-D) Total homogenates and synaptosomal fractions from human brains at different Braak stages were isolated from the frontal cortex **(A, B)** or hippocampus **(C, D)** and analysed by western blot with HT7 **(A, C)** or AT8 **(B, D)** antibodies. **(E-H)** Western blot quantification was performed and expressed as intensity of the bands per micrograms of protein (arbitrary unit/μg) loaded for the frontal cortex **(E, F)** and hippocampus **(G, H)** samples, respectively.

Supplementary Fig. 3

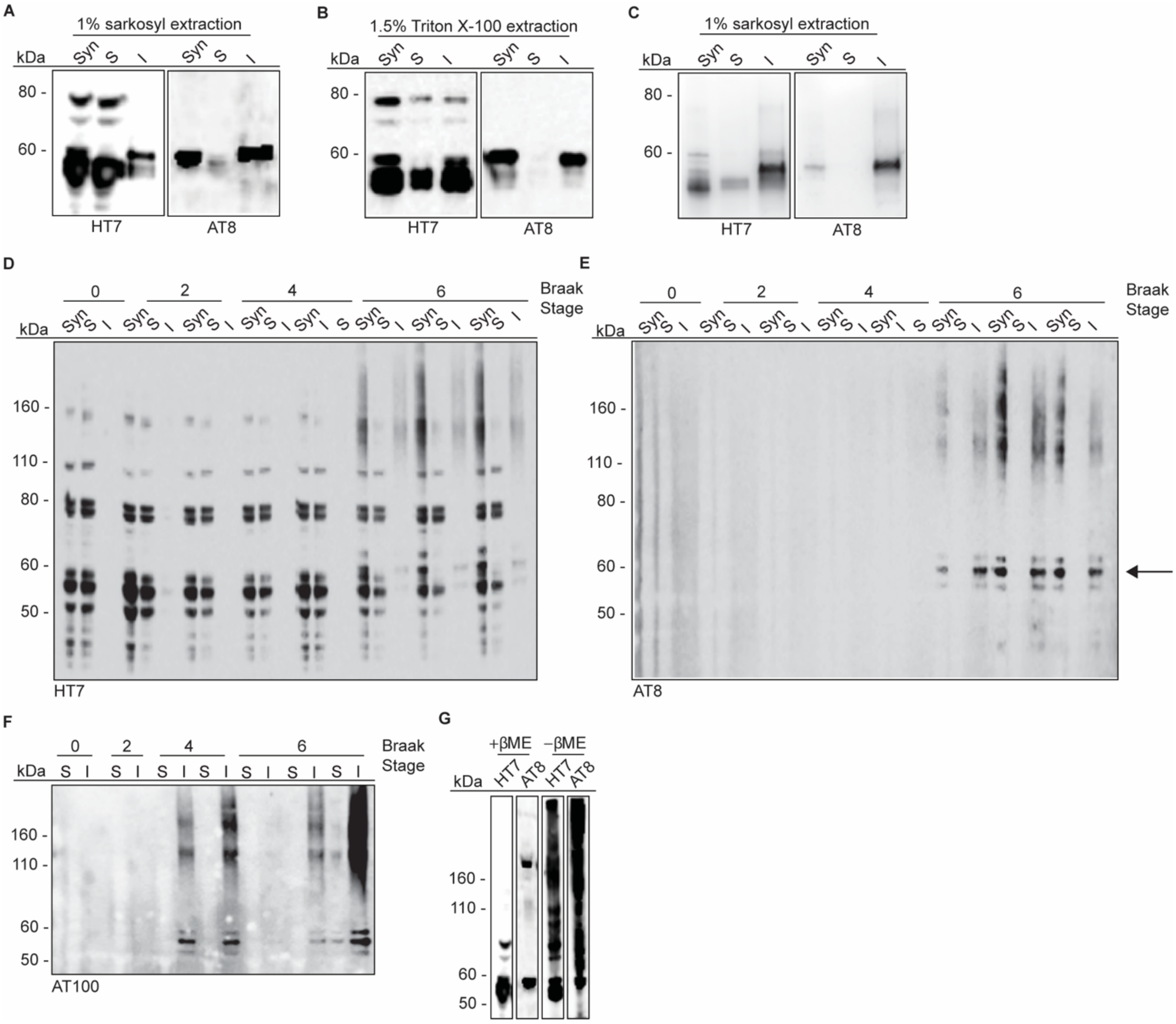

Supplementary Fig. 3. Detergent insoluble and aggregated tau in synaptosomes isolated from TgP301S and Tg4510 mice and human Alzheimer's disease brain.

(A and B) Synaptosomes (Syn) from TgP301S brain were treated with sarkosyl **(A)** or Triton X-100 **(B)** to separate soluble (S) and insoluble (I) tau fractions. With both detergents the insoluble fraction was enriched in a higher molecular weight HT7-positive band which was specifically recognised by the AT8 antibody (arrowheads). **(C)** Similar findings were obtained in Tg4510 synaptosomes treated with 1% sarkosyl. **(D and E)** Synaptosomes (Syn) from control and AD hippocampi were treated with sarkosyl to separate soluble (S) and insoluble fraction (I) and analysed in western blotting with HT7 **(D)** and AT8 **(E)** antibodies. Higher

molecular weight tau and aggregates recognised by the HT7 antibody were mainly seen in Braak stage VI brain synaptosomes and were enriched in the insoluble fractions. The AT8 antibody mainly recognised the higher molecular weight tau and the aggregates in Braak stage VI brain synaptosomes (arrow). **(F)** Similar results were seen in human AD synaptosomes with a different phospho-tau antibody (AT100). **(G)** Synaptosomes from TgP301S forebrain were prepared for SDS-PAGE with or without β-mercaptoethanol and analysed by western blotting with HT7 and AT8 antibodies. Large smears suggested the presence of tau aggregates under non-denaturating conditions. While all tau species were recognised by HT7, AT8 recognised only the higher molecular weight form of tau plus the aggregates.

Supplementary References

Bayes A, Collins MO, Galtrey CM, Simonnet C, Roy M, Croning MD, Gou G, van de Lagemaat LN, Milward D, Whittle IR, Smith C, Choudhary JS and Grant SG (2014) Human post-mortem synapse proteome integrity screening for proteomic studies of postsynaptic complexes. *Mol Brain* **7**:88.
